# Indolent natural killer-cell lymphoproliferative disorder of the gallbladder: a rare case report

**DOI:** 10.3389/fonc.2026.1649462

**Published:** 2026-04-28

**Authors:** Ya Jiang, Xiaofeng Xie, Ziran Gao, Wenmang Xu, Yuanyuan Wang

**Affiliations:** 1 Department of Pathology, 920th Hospital of the Joint Logistics Support Force of PLA, Kunming, China; 2 Department of Urology, The First Affiliated Hospital of Kunming Medical University, Kunming, China

**Keywords:** gallbladder, indolent natural killer-cell lymphoproliferative disorder, lymphomatoid gastropathy, NK-cell enteropathy, pathology

## Abstract

**Background:**

Indolent natural killer (NK)-cell lymphoproliferative disorder (iNKLPD) is a rare, recently defined neoplasm that is typically challenging to diagnose preoperatively. We report a rare case of iNKLPD arising in the gallbladder.

**Case presentation:**

A 61-year-old woman presented with right upper abdominal pain and a positive Murphy’s sign. Ultrasonography revealed wall thickening (0.6 cm) and a submucosal, tumor-like lesion at the gallbladder neck, along with several hyperechoic shadows suggestive of stones. Histopathological examination revealed coagulative necrosis in the mucosal lamina propria and diffuse infiltration of tumor cells into the muscular layer. The tumor cells were medium in size, with inconspicuous or small nucleoli. Immunohistochemically, the cells were positive for CD3, CD2, CD56, CD43, TIA-1, perforin, and granzyme B, and negative for CD5, CD20, CD79a, pan-CK, Bcl-6, CD10, MUM1, and cyclin D1. Both Epstein–Barr virus-encoded RNA *in situ* hybridization (EBER ISH) and T-cell receptor (TCR) gene rearrangement analysis by PCR were negative. Based on these findings, a pathological diagnosis of iNKLPD of the gallbladder was established. The patient remained recurrence-free at 36 months after surgery.

**Conclusion:**

We herein present a rare case of iNKLPD primarily occurring in the gallbladder. Given its atypical clinical and pathological features, iNKLPD requires careful distinction from other lymphomas or inflammatory lesions to prevent misdiagnosis. Furthermore, due to its indolent nature, accurate recognition is crucial to avoid the potential risk of overtreatment.

## 1 Introduction

Indolent natural killer-cell lymphoproliferative disorder of the gastrointestinal tract (iNKLPD-GI) is a rare, atypical NK-cell proliferation first described by Vega et al. and Takeuchi et al. (1, 2). Initially regarded as a reactive process under the former designations “lymphomatoid gastropathy” or “NK-cell enteropathy”, its neoplastic nature has been substantiated by the discovery of recurrent gene mutations, most notably in JAK3 (3). Consequently, iNKLPD-GI is now recognized as a distinct entity and has been incorporated into the fifth edition of the World Health Organization (WHO) classification of hematolymphoid tumors (4, 5). This disorder is characterized by a benign clinical course: individual lesions may regress spontaneously, new lesions appear only occasionally, and no progression to aggressive disease has been reported. Hence, it is designated as a “lymphoproliferative disorder”, with the qualifier “indolent” reflecting its favorable prognosis (3). To date, owing to its rarity, fewer than 70 cases have been documented (6). While typically confined to the gastrointestinal (GI) tract, lesions can also arise in extra-GI sites such as the esophagus, lymph nodes, nasopharynx, female urogenital tract, and gallbladder (6–8). Patients are often asymptomatic or present with non-specific gastrointestinal symptoms (9). Involvement solely in the gallbladder is exceedingly rare, as most previously reported cases with gallbladder involvement were part of multi-site disease (6, 9).

A literature search was performed on PubMed using the following keywords: 1) “NK-cell enteropathy” and/or “gallbladder”, 2) “lymphomatoid gastropathy” and/or “gallbladder, and 3) “benign” or “indolent NK-cell lymphoproliferation” and/or “gallbladder”. References from relevant original and review articles were also screened. Only one case, reportedly confined to the gallbladder, was identified (10); however, that report did not specify whether concomitant gastrointestinal lesions were present. As a rare entity, primary lymphoproliferative disorders of the gallbladder pose significant diagnostic challenges. These stem primarily from their clinical overlap with common gallbladder diseases and the complexity of pathological differentiation, often leading to misdiagnosis or diagnostic delay. Such errors can profoundly affect clinical management and patient prognosis. Pathologically, these lesions are particularly prone to being misdiagnosed as extranodal NK/T-cell lymphoma (ENKTCL). We herein report a consultation case of iNKLPD isolated to the gallbladder, which had been initially diagnosed as ENKTCL at an external institution. Timely and accurate recognition of this indolent disorder is crucial to avoid unnecessary overtreatment.

## 2 Case report

We report a consultation case from 2021 involving a 61-year-old woman who presented with a 3-day history of intermittent right upper quadrant pain of unknown etiology. The pain radiated to the right shoulder and back and was associated with nausea and vomiting. The vomitus was described as coffee-ground in appearance, although no frank hematemesis or melena was reported. Physical examination revealed jaundice of the skin and mucous membranes. The right upper abdomen exhibited mild tenderness, rebound tenderness, and localized muscle guarding. Murphy’s sign was positive.

**Figure 1:**
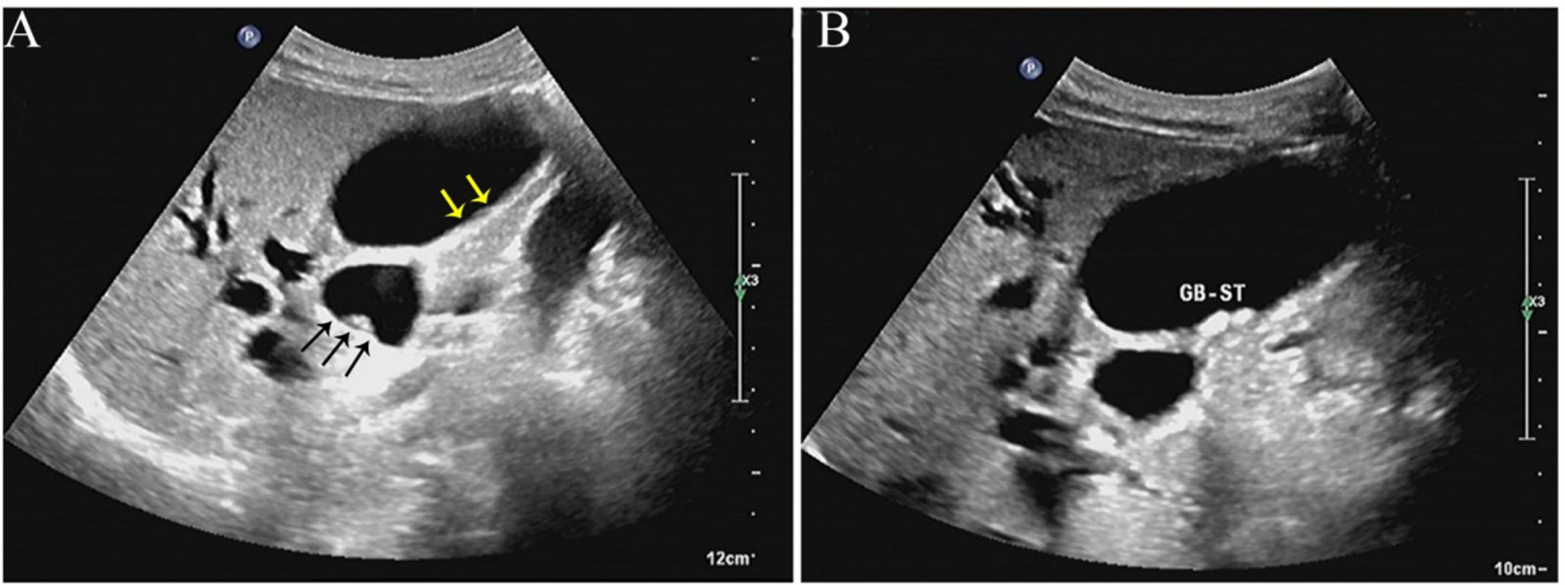
Ultrasonographic findings of gallbladder iNKLPD. **(A)** Transverse ultrasound image reveals focal, asymmetrical wall thickening of the gallbladder (yellow arrows). A corresponding hypoechoic, submucosal tumor-like lesion is identified at the gallbladder neck (black arrows). **(B)** A separate ultrasound image demonstrates multiple hyperechoic foci with posterior acoustic shadowing within the gallbladder lumen, consistent with cholelithiasis. iNKLPD, indolent natural killer-cell lymphoproliferative disorder.

Laboratory investigations on admission revealed markedly abnormal liver function tests: alanine aminotransferase (ALT) 237 IU/L, aspartate aminotransferase (AST) 105 IU/L, total protein (TP) 58.3 g/L, total bilirubin (TBIL) 102.8 μmol/L, direct bilirubin (DBIL) 73.34 μmol/L, alkaline phosphatase (ALP) 489 IU/L, indirect bilirubin (IBIL) 29.5 μmol/L, and γ-glutamyl transferase (γ-GGT) 545 IU/L. Tumor markers showed an alpha-fetoprotein (AFP) level of <1.1 ng/mL and CA19–9 at 40.15 U/mL. Abdominal ultrasound identified a submucosal tumor-like lesion in the gallbladder (**Figure 1A**), along with features consistent with acute cholecystitis, gallstones (**Figure 1B**), gallbladder wall thickening (0.6 cm) (**Figure 1A**), and bile duct stones associated with biliary dilation. Routine preoperative exams were otherwise unremarkable. The preliminary diagnoses were as follows: 1) gallbladder polyp with stones, 2) common bile duct stones with acute cholangitis, and 3) obstructive jaundice. Differential diagnoses included the following: 1) xanthogranulomatous cholecystitis and 2) gallbladder tumor. The imaging findings did not provide clear evidence of malignancy. Given clear surgical indications, the patient underwent laparoscopic cholecystectomy, choledochoscopy, and common bile duct exploration under general anesthesia 3 days after admission. Intraoperative findings included a thickened gallbladder wall, a 0.8-cm protrusion at the gallbladder neck, extensive adhesions between the gallbladder and the transverse colon, and a common bile duct diameter of 1.0 cm.

**Figure 2:**
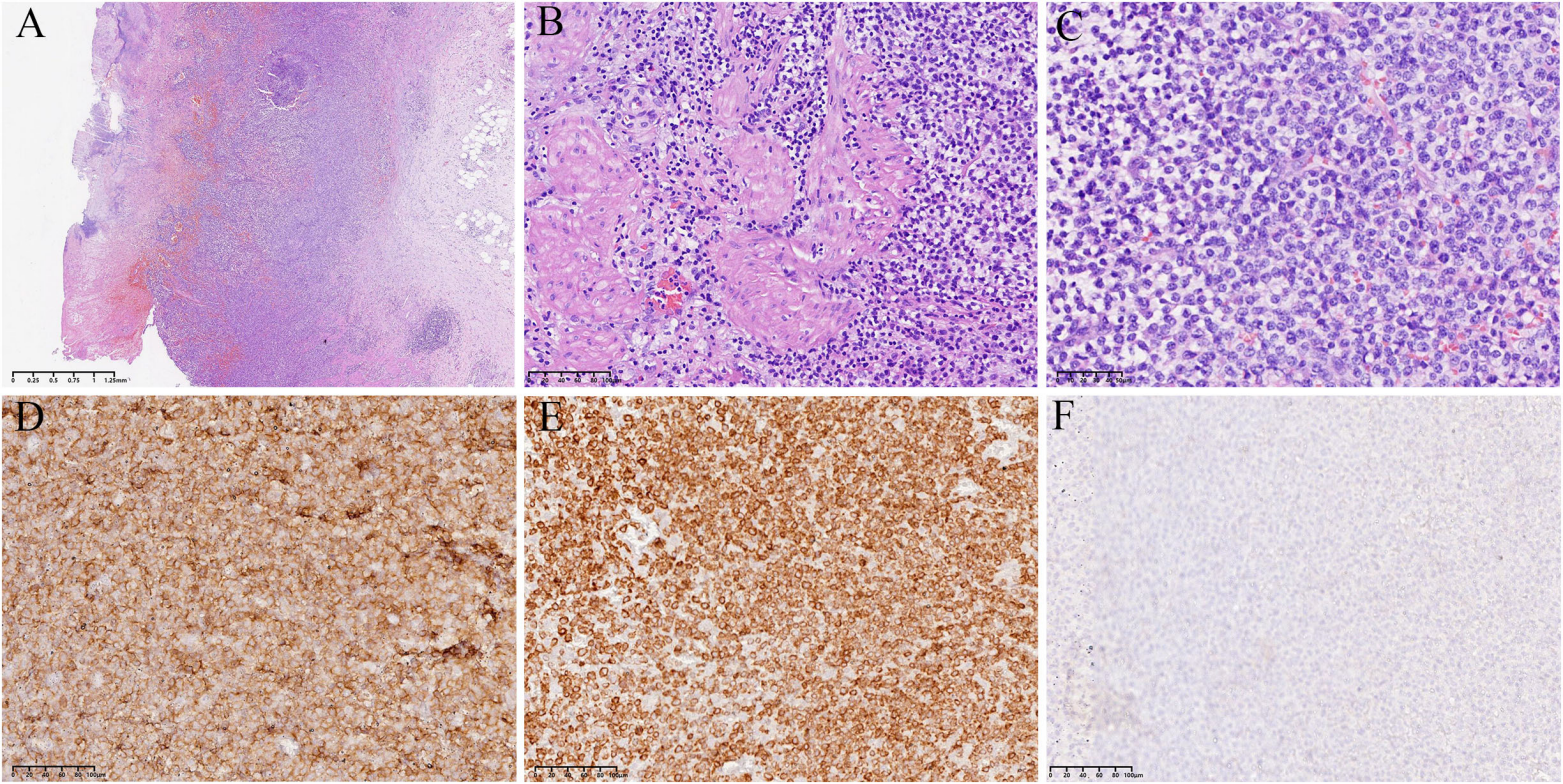
Pathological and immunophenotypic features leading to the diagnosis of iNKLPD of the gallbladder, revised from an initial misdiagnosis of ENKTCL. **(A)** Low-power view shows extensive mucosal necrosis. A dense infiltrate of small, blue, round lymphoid cells is present within the muscular layer, with focal areas suggestive of lymphoid follicle formation. The serosal layer exhibits mild inflammatory infiltration and edema (H&E staining). **(B)** Tumor cells diffusely infiltrate the muscularis propria; a subset demonstrates characteristic cytoplasmic clearing (H&E staining). **(C)** Higher magnification reveals a uniform population of medium-sized tumor cells with clear cytoplasm and occasional convoluted nuclei. Lymphocytic cuffing of small vessels is noted, without definitive angiocentric necrosis (H&E staining). **(D)** The lymphoid cells show strong positivity for CD3 (immunohistochemical staining, magnification ×200). **(E)** Strong and diffuse expression of CD56 is observed, confirming NK-cell differentiation (immunohistochemical staining, magnification ×200). **(F)** Epstein–Barr virus-encoded RNA (EBER) *in situ* hybridization is negative, a critical finding that excludes EBV-driven ENKTCL and supports the diagnosis of iNKLPD (magnification ×200). iNKLPD, indolent natural killer-cell lymphoproliferative disorder; ENKTCL, extranodal NK/T-cell lymphoma; EBV, Epstein–Barr virus.

Pathological examination of the gallbladder revealed extensive necrosis in the mucosal layer. At low magnification, a dense infiltrate of small, blue, round lymphoid cells was observed within the muscular layer, with focal areas suggestive of lymphoid follicle formation. The serosal layer exhibited mild inflammatory cell infiltration accompanied by edema (**Figure 2A**). The lymphoid cells infiltrated the muscularis propria; some displayed cytoplasmic clearing (**Figure 2B**). Tumor cells were uniform in size with clear cytoplasm, and occasional cells harbored convoluted nuclei (**Figure 2C**). Lymphocytic cuffing of small vessels was present, but no definitive angiocentric necrosis or vascular destruction was identified. Immunohistochemically, the lymphoid cells were positive for CD3 (**Figure 2D**), CD56 (**Figure 2E**), CD2, CD43, TIA-1, granzyme B, and perforin, while negative for CD5, CD20, CD79a, pan-CK, Bcl-6, CD10, MUM1, and cyclin D1. The Ki-67 proliferation index was high (approximately 80%). Epstein–Barr virus-encoded small RNA (EBER) *in situ* hybridization (ISH) was negative (**Figure 2F**). Additionally, polymerase chain reaction (PCR) analysis for T-cell receptor (TCR) gene rearrangement yielded a negative result. No enlarged lymph nodes involved by the disease were found in either the superficial or intra-abdominal regions. The original hospital had diagnosed ENKTCL without assessing Epstein–Barr virus (EBV) status. However, given the patient’s solitary gallbladder lesion and the negative EBER result, coupled with an indolent clinical course, the diagnosis was revised. The final diagnosis was iNKLPD of the gallbladder. The patient showed no signs of recurrence during 36 months of follow-up.

## 3 Discussion

INKLPD is a rare entity, and its occurrence as a solitary lesion in the gallbladder is exceedingly rare. The disease typically follows an indolent clinical course, with potential for spontaneous regression or persistence over many years, irrespective of therapeutic intervention. Previous reports of extragastric iNKLPD include five cases (aged 33 to 65 years) (summarized in **Table 1**), in which lesions were identified incidentally following cholecystectomy performed for chronic cholecystitis or biliary colic (10, 11). Among these cases, only one exhibited isolated gallbladder involvement, whereas the others presented alongside concurrent gastrointestinal lesions. Thus, the present case constitutes the second reported instance confined solely to the gallbladder. All documented cases of gallbladder iNKLPD manifested with abdominal pain, consistent with our patient, who was diagnosed following cholecystectomy for biliary colic. The biliary tract may represent a common extragastric site for this disease, with some studies proposing a potential origin from a tissue−specific subset of NK cells (12–15).

**Table 1:** Review of the literature on iNKLPD of gallbladder.

Case in literature	Initial diagnosis	Age, years	Sex	Presentation	Distribution of disease	Treatments/clinical follow-up	Immunophenotype, EBER ISH, and TCR PCR results
Present	ENKTL	61	Female	Intermittent right upper abdominal pain	Gallbladder	Laparoscopic cholecystectomy, management; 36 months; NED	CD3+/CD56+/CD2+/CD43+/granzyme B+, TIA-1+, perforin+/CD5−/CD20−/CD79a−/pan-CK−/Bcl-6−/CD10−/MUM1−/cyclin D1−/EBER ISH−/polyclonal TCR, Ki67 80%
Xia, D (10)	Natural killer-cell enteropathy	65	Female	Multiple episodes of biliary colic	Gallbladder	Laparoscopic cholecystectomy, management; 2 months; NED	CD3+/CD56+/granzyme B+, perforin+/CD5−/CD4−/CD8−/CD20−/CD43+/EBER ISH−, Ki67 (15%–20%)
Hwang et al. (11)	iNKLPD	33	Female	Abdominal pain, indigestion	Gallbladder and gastrointestinal tract	No further management; 36 months; NED	CD3−/CD56+/TIA, granzyme B, and/or perforin+/CD2+/CD5−/CD4−/CD8−/CD20−/CD30−/CD34−/CD68−/MPO−/EBER ISH−/polyclonal TCR/Ki67 N/A
Hongmei Yi (9)	iNKLPD	56	Female	Asymptomatic, then epigastric pain	Stomach, duodenum, ileum, colon, and gallbladder	Used mesalazine, thalidomide, prednisolone, and lenalidomide for treatment and management; 99 months; residual disease	cCD3+/CD56+/TIA, granzyme B+, perforin+/CD2+/CD7+/CD5−/CD4−/CD8−/CD20−/CD43+/EBER ISH−/polyclonal TCR/Ki67 10%–80%
Hongyun Chen (6)	iNKLPD-GI	56	Female	Epigastric pain	Stomach, duodenum, colon, and gallbladder	Cholecystectomy Lenalidomide, management; 33 months; residual disease	cCD3+/CD56+/TIA, granzyme B+/CD2+/CD7+/CD5−/CD4−/CD8−/CD20−/CD43+/EBER ISH−/TCR−/Ki67 70%
Hongyun Chen (6)	iNKLPD-GI	40	Male	Abdominal discomfort and bloody stools	Stomach, duodenum, ileum, colon, gallbladder, and urinary bladder	Cholecystectomy Glucocorticoids and tacrolimus, management; 31 months; residual disease	cCD3+/CD56+/TIA, granzyme B+/CD2−/CD7+/CD5−/CD4−/CD8−/CD20−/CD43+/EBER ISH−/TCR−/Ki67 30%

EBER, Epstein–Barr virus-encoded RNA; TCR, T-cell receptor gene rearrangement; +, positive; −, negative; NA, not available; NED, no evidence of disease; iNKLPD, indolent natural killer-cell lymphoproliferative disorder; ISH, *in situ* hybridization.

In the present case, the polypoid lesion of the gallbladder showed diffuse infiltration by atypical lymphoid cells with associated necrosis. These cells were positive for CD56 and cytotoxic markers and exhibited a high Ki-67 proliferation index, which initially prompted the original hospital to favor a diagnosis of ENKTCL without performing EBER ISH. However, classic ENKTCL histology is characterized by perivascular infiltration, an angiocentric/angiodestructive growth pattern, and extensive necrosis. The neoplastic cells are typically pleomorphic and cytologically atypical. Immunophenotypically, they express cytoplasmic CD3, CD56, and cytotoxic molecules (e.g., perforin, TIA-1, or granzyme B), and nearly all cases are positive for EBER. A review of the literature indicates that the most common lymphomas involving the gallbladder are diffuse large B-cell lymphoma, with occasional reports of follicular lymphoma. To date, no cases of ENKTCL primarily arising in the gallbladder have been documented. Histologically, our case did not demonstrate definitive angioinvasion by tumor cells. While necrosis was present, it may be attributable to overlying erosive inflammation rather than a primary feature of the neoplasm. Furthermore, the infiltrating lymphoid cells within the muscularis propria were relatively monomorphic, lacking the pronounced pleomorphism expected in ENKTCL. Critically, the EBER ISH performed at our hospital yielded a negative result. Regarding the Ki-67 proliferation index of up to 80%, this indeed reflects high proliferative activity. While iNKLPD is typically associated with a Ki-67 index below 50%, it can rarely reach up to 80% (6). Such focally high proliferative indices in iNKLPD may be related to the local microenvironment or reactive changes. Ultimately, considering the overall indolent clinical course (36 months without recurrence), the absence of EBER expression, and the lack of an angiodestructive pattern, the collective findings support a diagnosis of iNKLPD. We highlight this as a distinctive feature in our case and suggest that future studies further explore its significance.

A pivotal diagnostic feature of iNKLPD is the typical negativity for EBER. EBV is regarded as a key driver in the antigenic stimulation that precipitates malignant transformation and unchecked proliferation of NK cells. Current evidence underscores a strong association between ENKTCL and EBV infection, with positivity rates approaching 80%–100% in nasal cases (16), compared to lower rates (15%–50%) at other extranasal sites. Consequently, iNKLPD involving the gallbladder necessitates differentiation from other lymphoid tissue proliferative disorders. The differential diagnosis encompasses the following. 1) Aggressive NK-cell leukemia/lymphoma: This entity displays marked nuclear pleomorphism, with round or irregularly folded nuclei, condensed chromatin, and inconspicuous nucleoli. Frequent apoptotic bodies are present, and over 90% of cases are EBV-associated. 2) ENKTCL: As previously detailed, its hallmark features—including angiocentric/angiodestructive growth, coagulative necrosis, significant cytologic atypia, and strong EBER positivity—were absent in the present case. Distinguishing iNKLPD from the rare entity of EBV-negative ENKTCL can be challenging; however, as noted by Koo et al., the latter retains distinctively malignant histologic and immunophenotypic characteristics (17), in contrast to the indolent, often self-limiting nature of iNKLPD. 3) T-cell and B-cell lymphomas: Comprehensive immunophenotyping, coupled with the absence of clonal TCR and immunoglobulin heavy chain gene rearrangements, serves to reliably exclude these entities. This molecular profile is a key feature that distinguishes iNKLPD from clonal lymphomas of T- or B-cell origin. 4) Benign lymphoid hyperplasia: This reactive condition typically exhibits a polymorphous infiltrate comprising lymphocytes, plasma cells, and eosinophils. It is immunophenotypically negative for CD56 and demonstrates a mixed expression of B-cell (CD20 and CD79a) and T-cell (CD3 and CD5) markers without light chain restriction. Given its indolent clinical behavior, the accurate and timely recognition of iNKLPD is crucial to avoid unnecessary aggressive treatment.

From a molecular genetics perspective, a recent study by Xiao et al. first reported that 25%–33% of patients with iNKLPD harbor recurrent somatic JAK3 K563_C565del mutations, supporting its neoplastic rather than reactive origin (18). This finding further corroborates the tumorigenic nature of iNKLPD-GI. Although JAK3 mutations represent the most frequent genetic alteration in iNKLPD-GI, they are also commonly identified in aggressive NK- or T-cell lymphomas (19–22). These mutations lead to constitutive activation of downstream signaling pathways, particularly involving STAT3 and STAT5. Consistent with this mechanism, positive immunohistochemical staining for phosphorylated STAT5 and STAT3 has been demonstrated in biopsy specimens (9). Other reported mutations include BRAF V600E and KRAS T58I, which have previously been implicated in various hematologic malignancies such as myelodysplastic/myeloproliferative neoplasms and B-cell lymphomas. In the present case, being a consultation with a limited tissue sample, and given that the patient—upon understanding the indolent nature of the disease requiring only surveillance—declined further intervention, additional molecular testing was not pursued.

Given the indolent nature of the disease, the current lack of targeted therapies, and the absence of documented cases showing progression to lymphoma, a strategy of active clinical surveillance is recommended to avoid overtreatment. Conversely, for patients with symptomatic residual lesions, immunomodulatory agents—including glucocorticoids, thalidomide, and tacrolimus—have shown therapeutic efficacy in prior reports (6).

## 4 Conclusions

In conclusion, we present a rare case of iNKLPD occurring as a solitary lesion in the gallbladder. To date, only five such cases have been reported in the literature, with only one involving isolated gallbladder involvement. Although its incidence is exceedingly low, this entity carries considerable clinical significance. When localized to the gallbladder, iNKLPD can exhibit full-thickness wall infiltration—a distinctive feature compared to its usual presentation in the gastrointestinal tract. Moreover, the biliary tract may represent another common site of involvement alongside the GI tract. Due to its overlapping clinical and pathological features, iNKLPD necessitates careful differentiation from other lymphomas and inflammatory conditions to avoid misdiagnosis. Notably, the current WHO classification designates this entity as “gastrointestinal iNKLPD”; however, given its documented occurrence at extragastric sites, future revisions of the nomenclature may refine the terminology to more accurately reflect its anatomical distribution. Owing to its indolent clinical behavior, there is a tangible risk of overtreatment if the disorder is not recognized appropriately. While preliminary molecular findings suggest a potential link to the JAK–STAT signaling pathway, the precise pathogenic mechanisms driving iNKLPD remain unclear and warrant further investigation.

## 5 Strengths of the study

### 5.1 Clinical relevance

The manuscript describes a rare lymphoproliferative disorder with unusual anatomical localization, which may contribute to improved awareness among clinicians and pathologists.

### 5.2 Pathological documentation

The case includes detailed histopathological and immunohistochemical findings, including NK-cell markers (CD3, CD56, and cytotoxic proteins) and negative EBER results, which support the diagnosis.

### 5.3 Long follow-up period

The reported 36-month recurrence-free follow-up supports the indolent clinical behavior of the disease.

### 5.4 Educational value

The report highlights an important diagnostic pitfall, namely, the risk of misdiagnosing this entity as extranodal NK/T-cell lymphoma, which could lead to overtreatment.

## 6 Limitations of the study

### 6.1 Single case report

As expected for a case report, the conclusions are limited by the description of only one patient.

### 6.2 Limited molecular analysis

Being a consultation with a limited tissue sample, and given that the patient—upon understanding the indolent nature of the disease requiring only surveillance—declined further intervention, additional molecular testing was not pursued.

### 6.3 Incomplete imaging characterization

As this was a consultation case, the imaging materials provided by the patient were limited, precluding a more comprehensive radiological assessment.

## Data Availability

The original contributions presented in the study are included in the article/supplementary material. Further inquiries can be directed to the corresponding authors.
